# Recent Advances in Molecule Synthesis Involving C-C Bond Cleavage of Ketoxime Esters

**DOI:** 10.3390/molecules28062667

**Published:** 2023-03-15

**Authors:** Pu Chen, Huawen Huang, Qi Tan, Xiaochen Ji, Feng Zhao

**Affiliations:** 1 Key Laboratory for Green Organic Synthesis and Application of Hunan Province, Key Laboratory of Environmentally Friendly Chemistry and Application of Ministry of Education, College of Chemistry, Xiangtan University, Xiangtan 411105, China; 2 Hunan Provincial Key Laboratory for Synthetic Biology of Traditional Chinese Medicine, School of Pharmaceutical Sciences, Hunan University of Medicine, Huaihua 418000, China; 3 School of Chemistry and Chemical Engineering, Henan Normal University, Xinxiang 453007, China

**Keywords:** radical reactions, oxime ester, C-C bond cleavage, acylation, cyanoalkylation

## Abstract

The synthetic strategies of oxime derivatives participating in radical-type reactions have been rapidly developed in the last few decades. Among them, the N–O bond cleavage of oxime esters leading to formation of nitrogen-centered radicals triggers adjacent C–C bond cleavage to produce carbon-centered free radicals, which has been virtually used in organic synthesis in recent years. Herein, we summarized the radical reactions involving oxime N–O bond and C–C bond cleavage through this special reaction form, including those from acyl oxime ester derivatives and cyclic ketoxime ester derivatives. These contents were systematically classified according to different reaction types. In this review, the free radical reactions involving acyl oxime esters and cyclic ketoxime esters after 2021 were included, with emphasis on the substrate scope and reaction mechanism.

## 1. Introduction

The nitrogen-containing skeleton widely exists in a large number of natural products, active molecules and functional materials, and various commodities composed of them, such as food, drugs and various other materials that play crucial roles in human life [1,2,3,4,5,6]. The strategy involving nitrogen-centered radicals (NCRs), as a pragmatic strategy to construct a C–N bond rapidly, has always been one of the research hotspots of chemists in the last decades [7,8]. Although a large number of scientists have been committed to developing simpler and more effective paths for C–N bond construction, the development of a nitrogen-centered free radical strategy is still very limited, which is, as concluded by Zard [8], mainly hampered by “a dearth of convenient access to these species and a lack of awareness pertaining to their reactivity” [9].

**Scheme 1:**
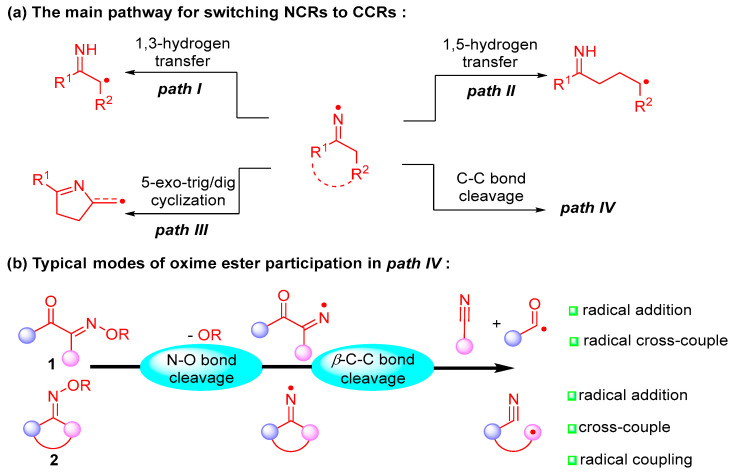
Main pathway switching for NCRs to CCRs.

Ketone-derivated oxime esters have been widely used in the field of organic synthesis [10,11,12,13]. Among them, one of the most important aspects is the application in the construction of nitrogen heterocyclic compounds [14,15]. In the past few decades, the implementation of transition metal catalysis with palladium, rhodium, ruthenium, cobalt, etc. for oxime N–O bond activation has been intensively reported, which exhibited excellent site selectivity and provided effective and convenient methods for the synthesis of aza- compounds [16]. For example, in 2022, the review of *O*-acyl ketoximes summarized by Huang’s group detailed the cases of the synthesis of nitrogen heterocyclic compounds under various transition-metal-based catalytic systems [16]. In recent years, free radical chemistry has been developed rapidly and has showed featured advantages, among which oxime esters were also widely used in free-radical-type reactions [17,18,19,20,21,22]. Typically, oxime ester derivatives generate imino radicals under the action of photocatalysts or transition metals, and then further convert into carbon-centered radicals (CCRs). The conversion from NCRs to CCRs mainly involves the following methods (Scheme 1a): (1) 1,3-hydrogen transfer process to give *α*-imidoyl carbon radicals (path I) [23,24]; (2) 1,5-hydrogen transfer process to generate a *γ*-imidoyl carbon radicals (path II) [25,26]; (3) intramolecular addition of an iminyl radical to a tethered alkene or alkyne in a 5-*exo*-trig or 5-*exo*-dig fashion (path III) [27,28,29]; and (4) intramolecular carbon–carbon bond cleavage to generate carbon radicals (path IV) [30,31,32].

Recently, there were many detailed reviews on these topics. In 2019, the Wang group disclosed a review on imino radical-involved reactions [33]. Subsequently, path II and III on photocatalytic cross-couplings via the cleavage of N–O bonds were described in the review by Fu and Zhu [34]. Recently, both the Zhou [31] and Samanta [32] groups presented detailed summaries of the mechanisms, as well as reaction forms, of the various reactions in which cyclic ketoximes are involved. In addition, numerous chemistry groups, including Zard [8], Chen [35], Xu [36], etc., have more or less summarized strategies for the above forms of nitrogen radical centers. However, the use of oxime esters as acylation reagents has only emerged in recent few years, and to our knowledge, there are no related reviews reported; therefore, we mainly focus on this topic to summarize related works. Since acyl oxime esters also belong to the path IV type, we have also added a summary of the work on cyclic ketoximes after 2021. The oxime ester compounds used in this reaction mode all have special structures, one with an *o*-diketone derivative and the other with a ring structure, which can be obtained by two-step conversion from the corresponding ketones. Meanwhile, they have a similar reaction mode (Scheme 1b) in that oximes undergo single electron transfer with a transition metal or photosensitizer to achieve N–O cleavage and afford imino radicals, which subsequently undergo *β-*C-C bond cleavage to generate acyl radicals or cyanoalkyl. The nitriles by-products are formed when using acyl oxime esters in reactions. The acyl radicals and cyanoalkyl radicals can be captured by various free radical receptors and participate in various forms of reactions [31,32,37,38,39]. In this review, various free radical reactions involving acyl esters and cyclic ketoxime esters are summarized, and they are classified according to different types of reactions, mainly including radical addition, cross-coupling and radical coupling.

## 2. Acyl Oxime Ester

### 2.1. Radical Addition

#### 2.1.1. Acyl Addition to Alkenes

**Scheme 2:**
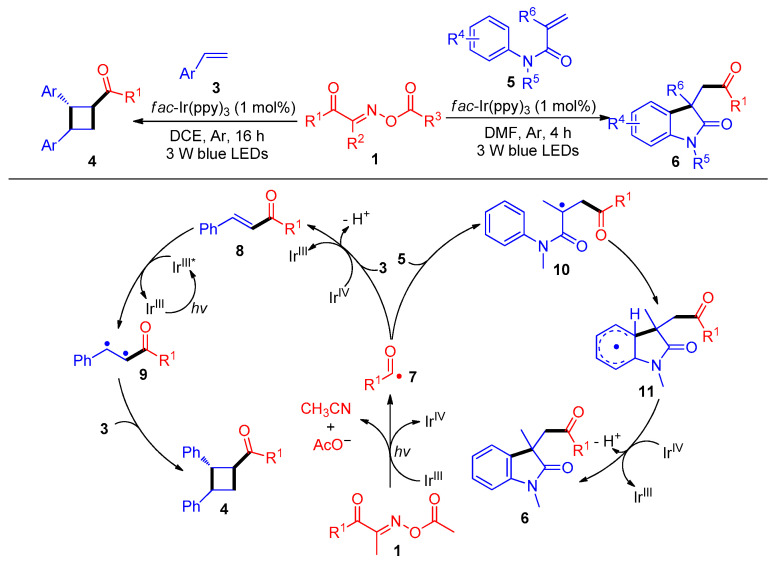
Visible-light-induced radical acylation/cyclization of acyl oxime esters with olefins.

In 2019, the Wu and Tung group demonstrated a groundbreaking example of acylation/cyclization of Michael acceptors with acyl radicals derived from acyl oxime esters **1** with C–C bond cleavage. Hence, cyclobutyl ketone derivatives **4** and acylated oxindoles **6** were obtained using *fac*-Ir(ppy)_3_ as a photosensitizer at room temperature (Scheme 2) [40]. The mechanism studies show that the acyl oxime esters **1** are first reduced by a Ir^III^ catalyst under 3 W blue-light conditions, and then the acyl radicals **7** are produced by C–C bond cleavage. On the one hand, the acyl radical **7** is captured by styrene **3** and oxidized by Ir^IV^ to remove a proton to produce enone **8**, which is sensitized by Ir^III*^ to further react with another styrene **3** to lead to cross [2 + 2] cyclization and obtain acylated cyclobutanones derivatives **4**. Alternatively, acyl radicals **7** could also add to the C–C double bond of the acrylamides **5** to afford structurally useful acylation oxindole skeletons **6**.

**Scheme 3:**
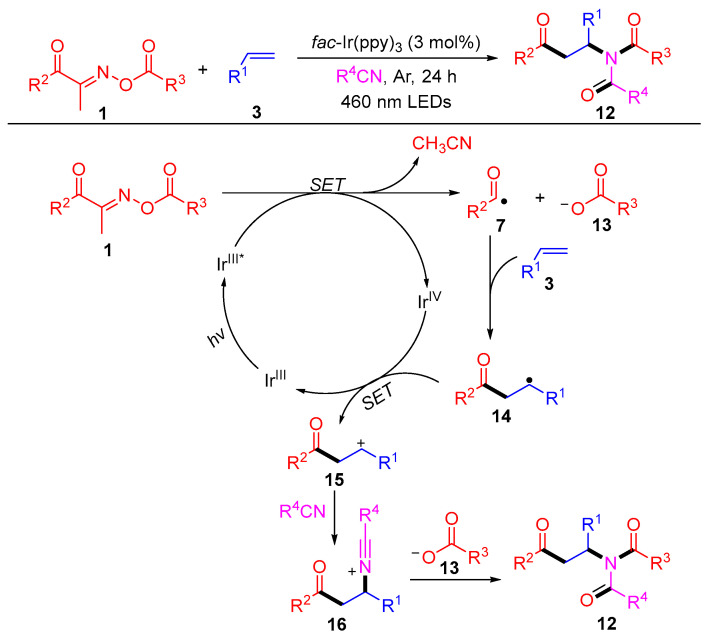
Visible-light-induced radical difunctionalization of acyl oxime esters with styrenes and nitriles.

In the same year, this group reported a novel three-component difunctionalization reaction of acyl oxime esters, olefins and alkane nitriles, which generated a series of *β*-carbonyl imides **12** with excellent yields (Scheme 3) [41]. The isotope tracking experiment and control experiments revealed the efficient intermolecular reorganization of oxime esters into styrene with the aid of solvent exchange. A possible reaction mechanism is shown in Scheme 3. First, the acyloxime esters **1** are cleaved to nitrile, carboxylate anions **13** and acyl radicals **7** by a single electron transfer (SET) process with the excited state Ir^III*^, which is generated from Ir^III^ under blue-light irradiation. Then, a SET occurs between intermediate **14**, which is produced by the addition of an acyl radical **7** to the olefin **3** and Ir^IV^ to complete the cycle of catalyst Ir and produce the carbocation **15**. The intermediate **16**, produced by the nucleophilic attack of nitrile to cation **15**, undergoes a Mumm rearrangement with carboxylate anions **13** to obtain the amide products **12**.

**Scheme 4:**
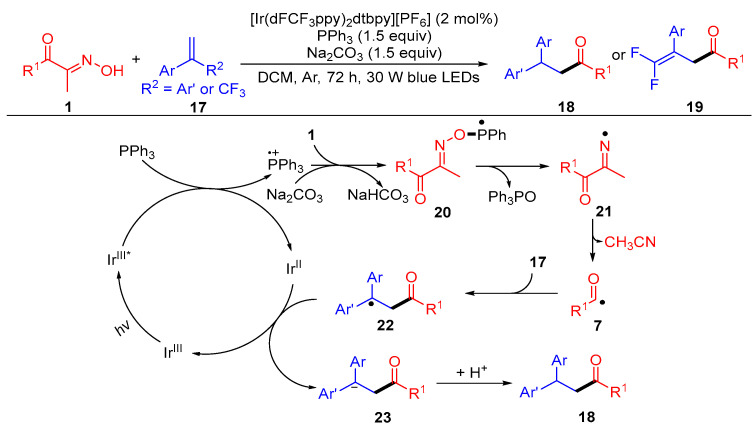
Visible-light-induced hydroacylation of acyl oximes with alkenes.

A photocatalytic hydroacylation of alkenes with acyl oximes for the synthesis of valuable ketones was developed by Yang et al. (Scheme 4) [42]. When CF_3_-attached styrenes were used, a E1cB-type fluoride elimination pathway was proposed for the obtainment of the 1,1-difluoroolefin products **19**. In this difunctionalization reaction, the triphenylphosphine radical cation generated by the reaction of the excited catalyst Ir^III*^ with triphenyl phosphine combines with the nucleophile acyl oximes **1** to obtain the phosphorus radical intermediate **20**. The N–O bond cleavage of the intermediate **20** gives the iminyl radical **21** via *β*-scission. The imino radical **21** undergoes C–C bond scission to give acyl radical **7** and a molecule of acetonitrile. The acyl radical is added to alkene **17** and reduced by Ir^II^ to get the carbanion intermediate **23**, with the following protonation to generate the desired product **18**.

**Scheme 5:**
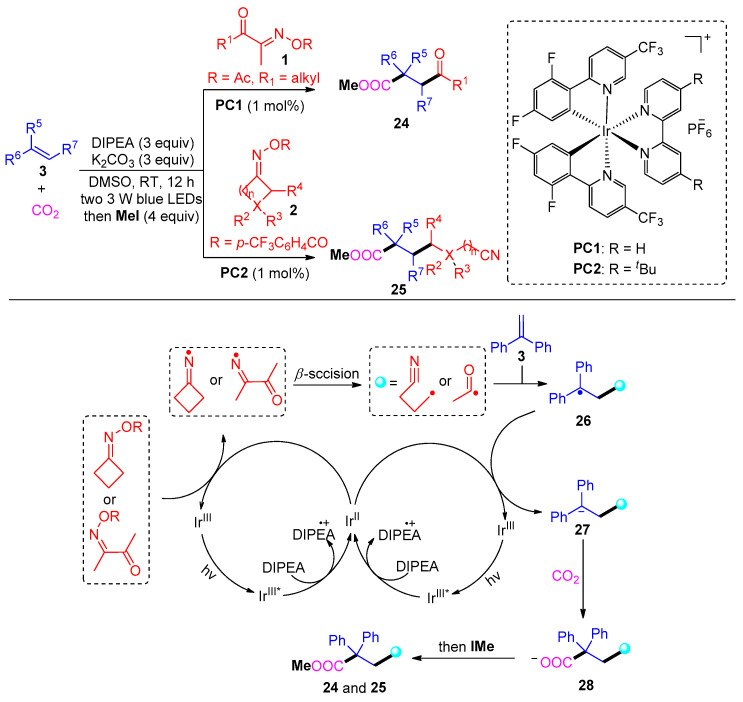
Visible-light photoredox-catalyzed dicarbofunctionalization of styrenes with oxime esters and CO_2_.

In 2021, Sun and co-workers developed a distinctive carbonylation approach using Ir and DIPEA to access *γ*-keto acids **24** and cyanocarboxylic acids **25** (Scheme 5) [43]. Mechanistically, this reaction involves the photocatalytic radical addition of acyl radicals and cyanoalkyl radicals to aromatic olefins and then carbon dioxide capture. The valuable dicarbofunctionalization tolerates a wide range of cyclic ketoxime substrates; however, only aliphatic acyl oxime esters proceed smoothly to afford corresponding *γ*-keto ester products. 

**Scheme 6:**
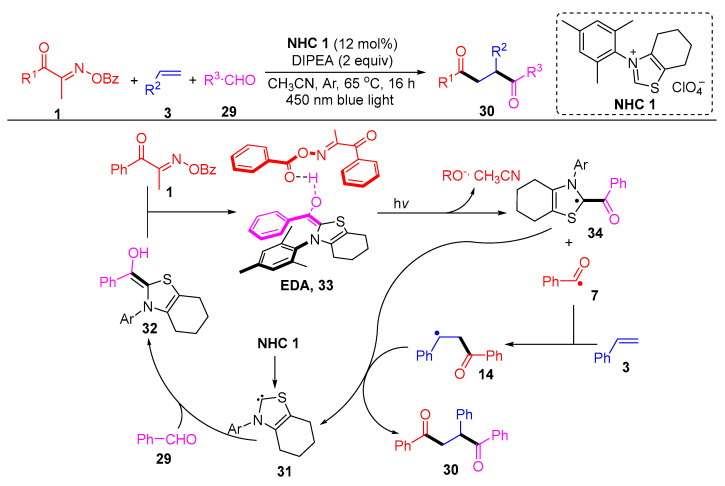
*N*-heterocyclic carbene-photocatalyzed 1,2-diacylation of alkenes with acyl oxime esters and aldehydes.

Larionov’s group described a *N*-heterocyclic, carbene-photocatalyzed, three-component regioselective 1,2-diacylation of alkenes, using acyl oxime esters and aldehydes as two different acylating agents (Scheme 6) [44]. In particular, the authors demonstrated the mechanism by density functional theory (DFT) and time-dependent-DFT (TD-DFT), where an EDA intermediate is probably formed in the diacylation, which initiates photoexcitation to mediate charge transfer. The EDA intermediate **33**, formed by the complexation of the Breslow intermediate **32** with the acyl oxime ester **1**, is excited under light conditions and subsequently cleaved to remove BzO^-^ and acetonitrile to give the intermediate **34** and acyl radical **7**. The intermediate **14** produced by the addition of the acyl radical **7** to the olefin **3** couples with the intermediate **34** and then decomposes to a diketone product **30** and carbene **31**.

**Scheme 7:**
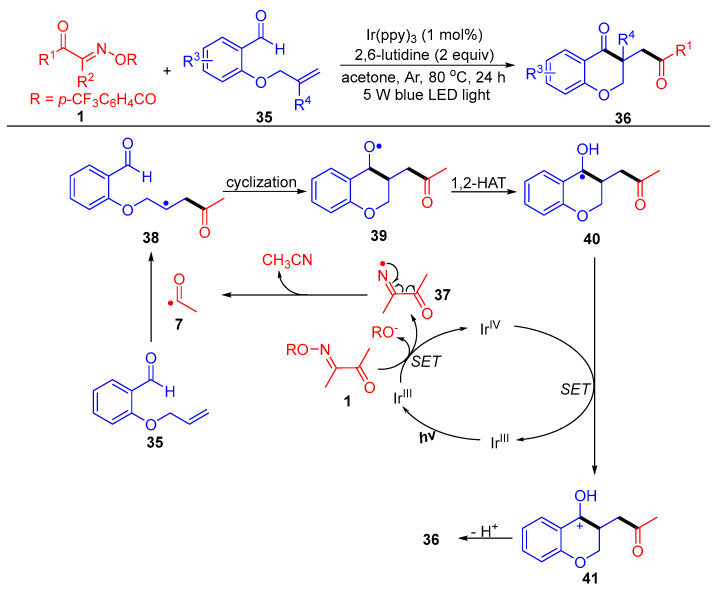
Visible-light-induced acylation of acyl oxime esters with 2-allyloxy benzaldehydes.

Liu and Huang’s group reported an intriguing route to 3-acyl-substituted chroman-4-one derivatives **36**, which involves SET reduction of acyl oxime esters **1** by *fac*-Ir(ppy)_3_ under thermal and light conditions (Scheme 7) [45]. In this functionalization reaction, the acyl radical generated from the acyl oxime ester first adds to the carbon double bond of the 2-allyloxy benzaldehydes **35**, followed by intramolecular cyclization and 1,2-HAT (hydrogen atom transfer) to obtain the alcohol radical intermediate **40**. Finally, SET oxidation by Ir^IV^ and deprotonation occur to obtain the target products **36**.

**Scheme 8:**
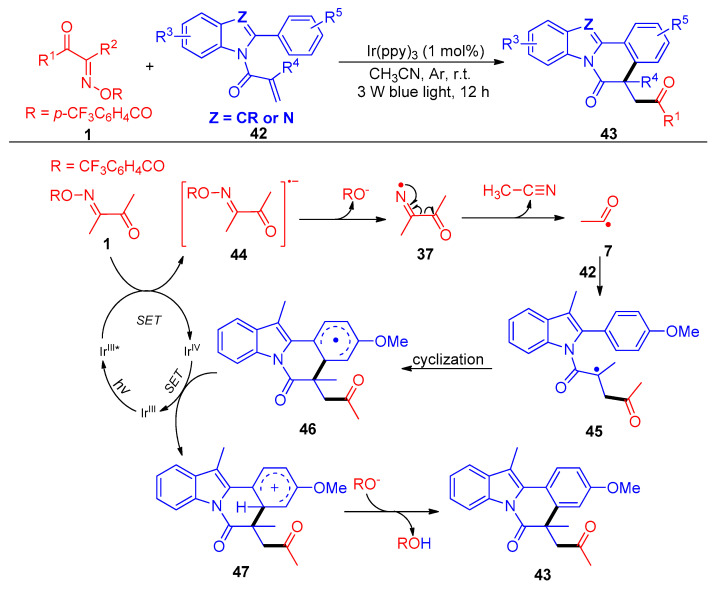
Visible-light-induced acylation of acyl oxime esters with reactive olefins.

Another acylation/cyclization strategy mediated by a photocatalytic nitrogen-centered radical of acyl oxime esters **1** with activated acrylamides **42** was reported by Liu and Huang’s group (Scheme 8) [46]. The authors screened a variety of acyl oximes with different leaving groups, such as 4-CF_3_C_6_H_4_CO, 4-NO_2_C_6_H_4_CO, C_6_F_5_CO, CF_3_CO, etc., and achieved up to 95% yields with 58 synthetic examples of acylated transformations. The fragmentation of one C–C bond and one N–O bond and the formulation of two new C–C bonds were carried out in one-pot conditions with a low catalyst loading (1 mol%). This protocol represents a simple and green road for the synthesis of acylated indolo/benzimidazo-[2,1,a]isoquinolin-6(5*H*) ones.

**Scheme 9:**
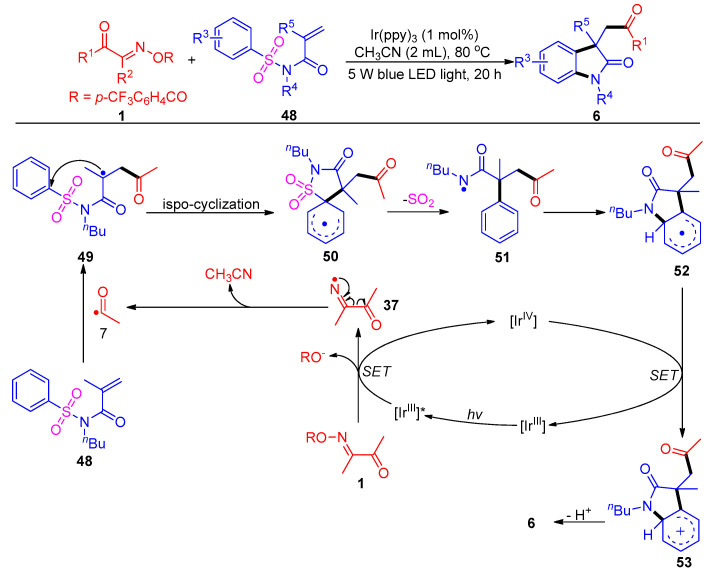
Visible-light-induced acylation of acyl oxime esters with *N*-(arylsulfonyl)acrylamides.

Most recently, the same group uncovered a new acylation reaction of acyl oxime esters with activated *N*-sulfonyl acrylamides **48,** with a broad substrate scope and good functional group compatibility (Scheme 9) [47]. This method provides an effective nitrogen center-mediated approach for the generation of acyl radical intermediates to access acylated oxindole **6**. This acylation transformation proceeds through a normal Smiles rearrangement process that involves cascade intramolecular *ispo*-cyclization (**50**), de-SO_2_ (**51**) and re-cyclization (**52**).

#### 2.1.2. Acyl Addition to Alkynes

**Scheme 10:**
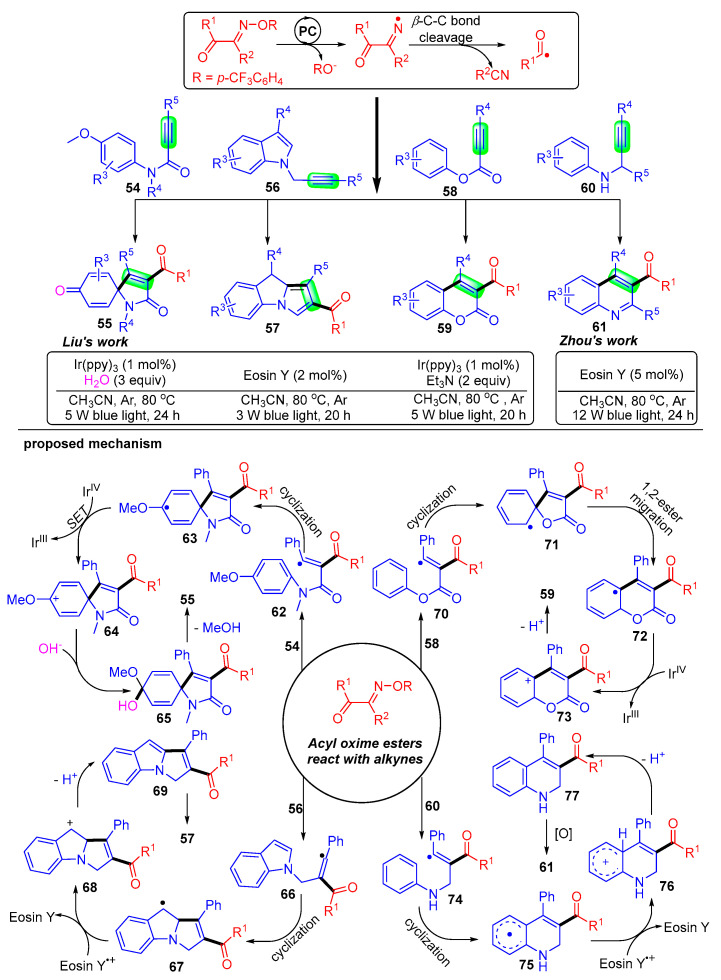
Visible-light photoredox-catalyzed acylation of alkynes with acyl oxime esters.

The heterocyclic skeleton presents in commercial drugs and many naturally occurring compounds; thus, the development of convenient and environmentally friendly methods for the construction of this structural motif is an important and promising research field [48,49,50]. Alkynes with specific substituents, such as alkyne amides [51,52,53], *N*-propylindoles [54,55,56], alkyne esters [57,58], alkyne amines [59,60,61], etc., are often used as radical acceptors to construct potential heterocyclic compounds by tandem cyclization. Recently, Liu and Zhou have independently developed a multitude of innovative photocatalytic, nitrogen-centered, radical-mediated acylation strategies of reactive alkynes using acyl oxime esters as acyl sources (Scheme 10) [37,39,62,63]. 

Liu’s group pioneered a radical addition strategy of acyl oxime esters **1** with activated alkynes **54** to construct a series of 3-acylated spiro [4,5]trienones derivatives **55** [37]. The acyl radical generated by the C–C bond breakage of acyl oxime esters attacks the carbon–carbon triple bond of propiolamides **54** to give the radical intermediate **62**. The intermediate **62** undergoes intramolecular *ispo*-cyclization and is oxidized to carbocations **64** by Ir^IV^, followed by the combination with hydroxide anion produced in water to obtain the intermediate **65**. Finally, a methoxy anion and hydrogen ion are removed to obtain the expected trienone product **55**.

Subsequently, Liu’s group developed two pragmatic acylation strategies using *N*-propynylindoles **56** [63] and alkynoates **58** [62] under photoexcitation conditions. A large quantity of acylated pyrrolo[1,2-*a*]indole **57** and coumarin **59** derivatives were constructed, and interestingly, both the alkanoyl and aryl groups were well adapted. These studies further developed the application of acyl oxime esters and were of great importance for the development of free radical synthetic chemistry.

Comparably, Zhou’s group has achieved a series of constructions of 3-acyl quinoline skeleton **61** under photocatalytic conditions with *N-*propargyl aromatic amines **60** as radical acceptors [39]. The mechanism of this acylation reaction states that acyl radicals add to the carbon–carbon triple bond of *N-*propargyl aromatic amines **60** for intramolecular cyclization and then proceed to dehydroarylation to give the final quinoline product **61**.

### 2.2. Radical Cross-Couple

**Scheme 11:**
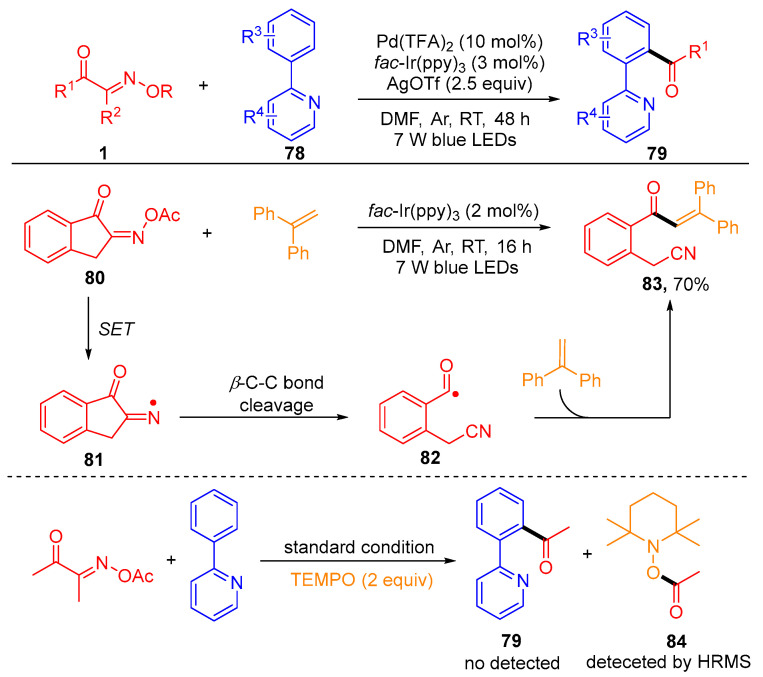
Photoredox/palladium-catalyzed C-H acylation of 2-arylpyridines with acyl oxime esters.

Recently, the development of the photoredox/palladium-catalyzed C-H acylation of 2-arylpyridines **78** with acyl oxime esters **1** using *fac*-Ir(ppy)_3_ as a photosensitizer was reported by the Chen group (Scheme 11) [64]. In order to thoroughly study the reaction mechanism, the author carried out some control experiments, including the radical clock reaction using benzocycloketoxime ester **80** with ethene-1,1-diyldibenzene under standard conditions and TEMPO as a radical inhibitor to test the activity of the reaction. The generation of the target product **83** and the detection of the free radical capture product **84** by the HRMS indicate that this acylation reaction contains a free radical mechanism.

**Scheme 12:**
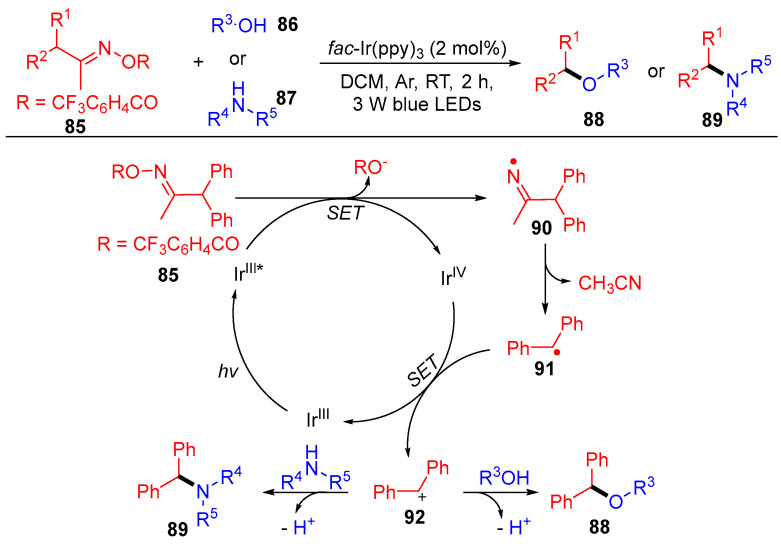
Visible-light-induced coupling of benzyl oxime esters with O- and N- nucleophiles.

In addition to the above acyl oxime esters, Wu’s group reported the benzyl oxime esters **85** with the same reaction mode, i.e., a single electron transfer followed by carbon–carbon bond cleavage to produce the corresponding radical (Scheme 12) [38]. The resulting benzyl radicals **91** are further oxidized to benzyl carbocation **92**, which are then coupled with O and N-nucleophilic reagents to access the target benzyl ethers **88** and benzylamines **89**. This benzylation strategy well tolerates various functional groups under mild conditions, where a wide range of nucleophilic substrates, such as primary and secondary alcohols, amines and even H_2_O, worked smoothly.

## 3. Cyclic Ketoxime Esters

### 3.1. Radical Addition

#### 3.1.1. Ring Opening and Addition to Alkenes

**Scheme 13:**
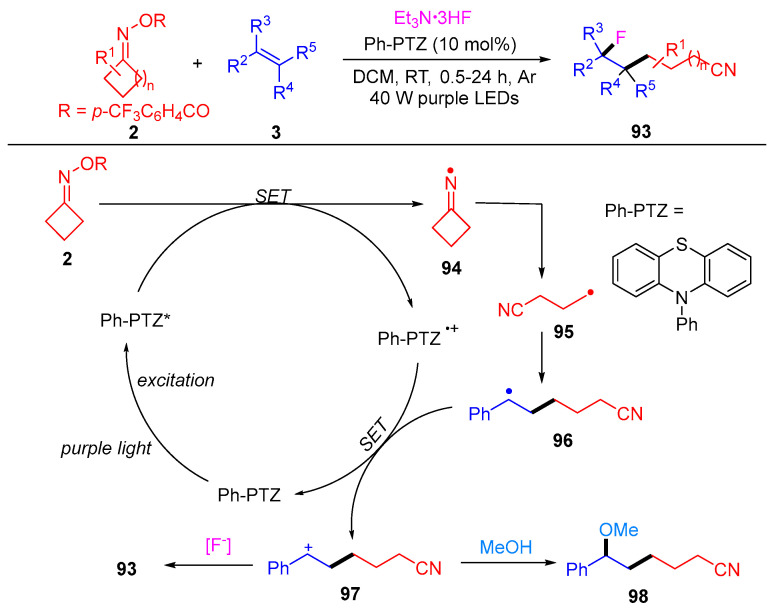
Visible-light-induced cyanoalkylfluorination of alkenes with cycloketone oxime esters and fluoride.

In 2021, Chen and co-workers reported a difunctionalization synthesis of cyanoalkylfluorinated products that exploits a 10-phenyl-10*H*-phenothiazine (Ph-PTZ) as a photosensitizer approach (Scheme 13) [65]. The three-component cyanoalkylfluorination is initiated by the excitation of Ph-PTZ by 40 W purple LEDs. The cyanoalkyl radical was obtained by the C–C bond cleavage of the imino radical **94** generated by the single electron transfer between the excited-state Ph-PTZ* and the cycloketoxime ester. Oxidation of the intermediate **96** by Ph-PTZ^•+^ delivers the carbocation intermediate **97** and Ph-PTZ. The resulting intermediate is then attacked by nucleophilic substances, such as F^−^ and alcohols, to produce corresponding products (**93** and **98**). This strategy provides a transition-metal-free nitrogen-centered radical pathway for the construction of fluorocyanoalkylation derivatives.

**Scheme 14:**
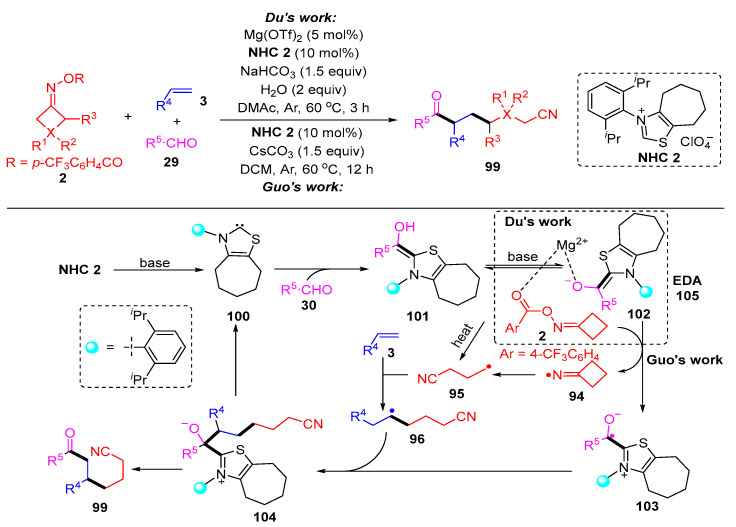
*N*-heterocyclic carbene-catalyzed radical acylation/cyanoalkylation of alkenes with cycloketone oxime esters and aldehydes.

In the same year, Du [66] and Guo [67] independently reported *N*-heterocyclic, carbene-catalyzed, three-component acylation/cyanoalkylation of alkenes with cyclic ketoxime esters and aldehydes (Scheme 14). The Du group suggested that this radical relay process relied on the SET achieved by the EDA complex **105** bound by Mg, the Breslow intermediate and oxime esters. The EDA complex **105** was subjected to thermally controlled SET and cleaved off the ester group to give the cyanoalkyl radical **95** and the intermediate **103**. However, Guo’s group believed that it was the acyl oxime ester that directly undergoes SET with the intermediate bound by the aldehyde and *N*-heterocyclic carbene to remove the ester group to form the butyl cyano radical and intermediate **103**. Subsequently, the addition of cyanoalkyl radicals **95** to olefins **3**, followed by the radical-coupling with the intermediate **103**, occurred to obtain the intermediate **104**, which was further transformed to the final product **99** with the regeneration of the NHC for the next catalytic cycle.

**Scheme 15:**
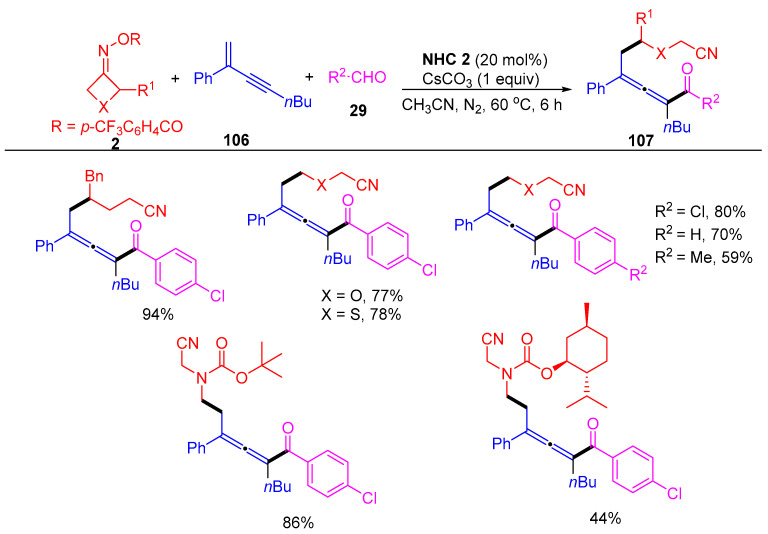
*N*-heterocyclic carbene-catalyzed 1,4-alkylcarbonylation of 1,3-enynes with cycloketone oxime esters and aldehydes.

In addition, Du’s group developed *N*-heterocyclic carbene-catalyzed 1,4-alkylcarbonylation of 1,3-enynes **106** with aldehydes **29** and different alkyl radical precursors, such as CF_3_I, alkyl halides, cycloketone oxime esters and redox-active esters derived from aliphatic carboxylic acids, affording a series of *tetra*-substituted allenyl ketones with excellent yields [68]. Among them, a variety of cyanoalkylated and acylated bianenone derivatives **107** were obtained in yields of 44–94% when cyclic ketoxime esters **2** were involved in this *N*-heterocyclic carbene-catalyzed system (Scheme 15).

**Scheme 16:**
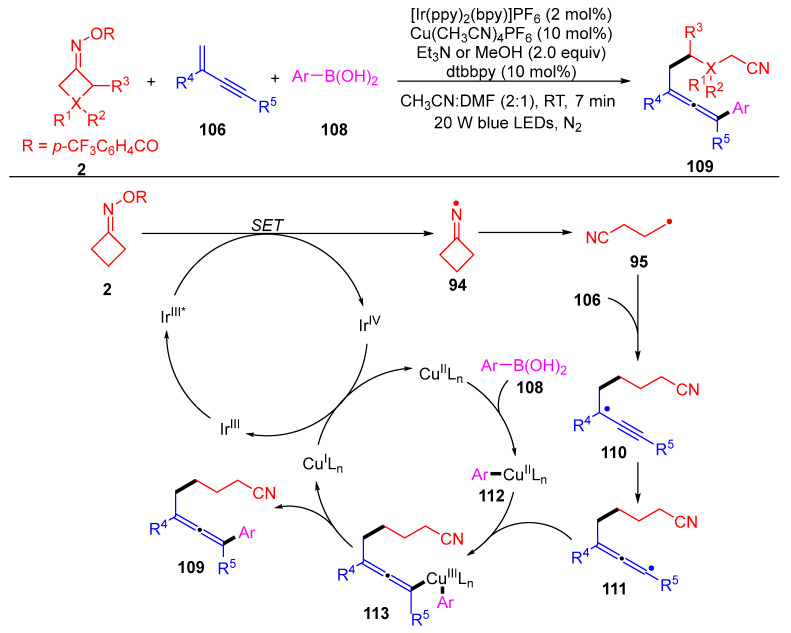
Photo/Cu co-catalyzed 1,4-cyanoalkylarylation of 1,3-enynes with cycloketone oxime esters and boronic acids.

Guo and co-workers reported 1,4-cyanoalkylarylations of 1,3-enynes **106** and aryl boronic acids **108** with a range of cyclobutanone oxime esters **2** to furnish multiple functionalizatized allenes **109**. In the presence of [Ir(ppy)_2_(bpy)]PF_6_ and Cu(CH_3_CN)_4_PF_6_, the reaction completed within 7 minutes to reach 80% yield (Scheme 16) [69]. Cyclic ketoxime esters **2** were smoothly disintegrated and ring-opened to cyanoalkyl radicals with the help of the Ir^III*^ catalyst, followed by the addition to the olefin of 1,3-enynes to obtain the radical intermediate **110**, which underwent a radical shift to give the allene radical species **111**. The oxidative addition of Ar-Cu(II) in situ formed by the transmetallation of the Cu(II) species with aryl boronic acids **108** to the diolefin radical **111** gives the intermediate **113**, which undergoes reductive elimination to provide the target *tetra*-substituted allene **109** and the Cu(I) species.

**Scheme 17:**
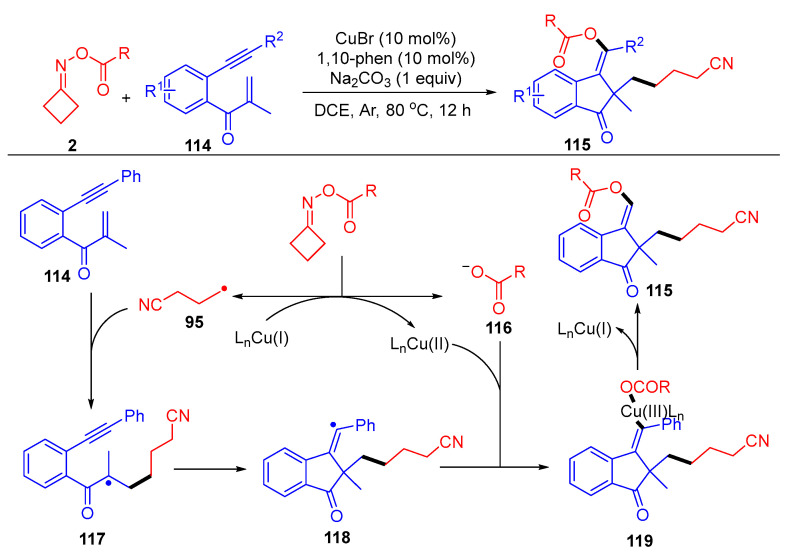
Cu-catalyzed difunctionalization of 1,6-enynes with cycloketone oxime esters.

Jiang et al. have established the 100-percent atom utilization, radical, tandem difunctionalization of 1,6-enynes **114** with cycloketone oxime esters **2** for the synthesis of 1-indanones bearing an all-carbon quaternary center by using CuBr as the catalyst, 1,10-phen as the ligand and Na_2_CO_3_ as the base (Scheme 17) [70]. The alkenyl radical intermediate **118** obtained by cyclization after attacking the alkyne **114** by the cyanoalkyl radical **95** reacted with the carboxylate anions **116** and Cu(II) species, producing all-atom utilization target products **115**.

**Scheme 18:**
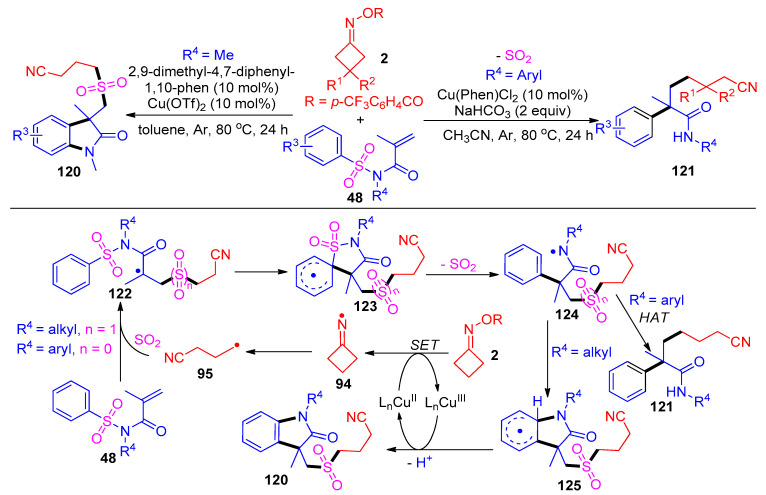
Cu-catalyzed difunctionalization of *N*-sulfonylated acrylamides with cycloketone oxime esters.

In 2021, a splendent method toward cyanoalkylsulfonylated oxindoles **120** and cyanoalkyl amides **121** was developed by Liang and co-workers (Scheme 18) [71]. Interestingly, the properties of substituent groups on the nitrogen atom in activated alkenes **48** resulted in different products. In particular, when the R^4^ = Me, oxindoles **120** were accessed. More surprisingly, the cyanoalkyl radical in the cyclization process could capture SO_2_ from the *N*-sulfonylated acrylamides **48**, thus realizing cyanoalkylation/sulfonylation without an additional sulfur source. On the other hand, when *N*-arylacrylamides (R^4^ = aryl) were subjected to the system, hydrogenated cyanoalkyl amides were afforded.

**Scheme 19:**
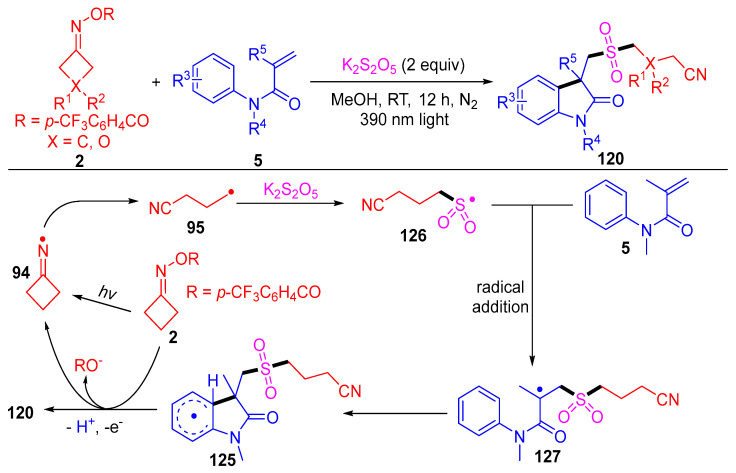
Photoinduced cyanoalkylsulfonylation of *N*-arylacrylamides with cycloketone oxime esters.

A cascade cyanoalkylation/sulfonylation/cyclization of *N*-arylacrylamides **5** was reported by Gui’s group, which accessed a range of cyanoalkyl sulfonylated oxindole products **120** in good yields (Scheme 19) [72]. Compared to other strategies, this ring-opening sulfonylation transformation avoids the usage of catalysts and oxidants and requires only a 390 nm purple light source to proceed smoothly. The light irradiation was proposed to enable the N–O bond cleavage of ketoximes in the initial step.

**Scheme 20:**
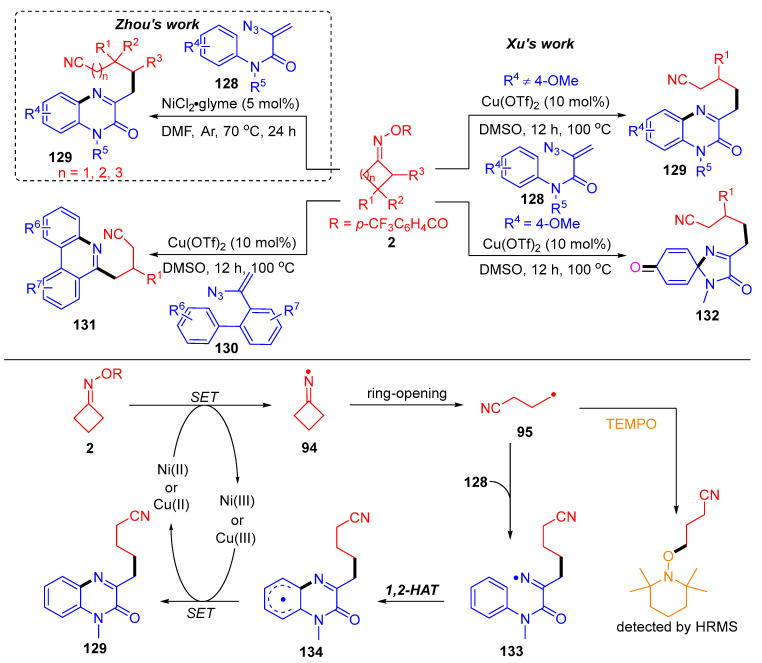
Nickel- or copper-catalyzed cyanoalkylation of vinyl azides with cycloketone oxime esters.

Nitrogen-containing heterocycles, especially phenanthridine and quinoxaline derivatives, have been applied extensively in drugs, materials and organic synthesis [73,74]. In 2021, Zhou et al. developed nickel-catalyzed, difunctionalization, oxidative annulation reactions of vinyl azides **128** and cycloketone oxime esters **2** to prepare cyanoalkyl containing quinoxalin-2(1*H*)-ones **129** (Scheme 20) [73]. One of the highlights of the strategy is that five- and six-membered cyclic ketoxime esters could also react to obtain ring-opening products in moderate yields. In the same year, a similar transition metal-catalyzed system for cyanoalkylation and cyclization of activated alkenes and cyclic ketoxime esters **2** was developed by Xu, Kong and co-workers (Scheme 20) [74]. Compared to Zhou’s work, this reaction replaces the nickel catalyst with a metallic copper catalyst and extends it to 2-(1-azidovinyl)-1,1′-biphenyl derivatives **131**. Further, spirocyclic ketone products **132** were obtained when 2-azido-*N*-(4-methoxyphenyl)acrylamides were used as the substrate. While the origin of the ketone oxygen atom has not been further verified by the authors, it usually comes from the water in the solvent in such a reaction.

**Scheme 21:**
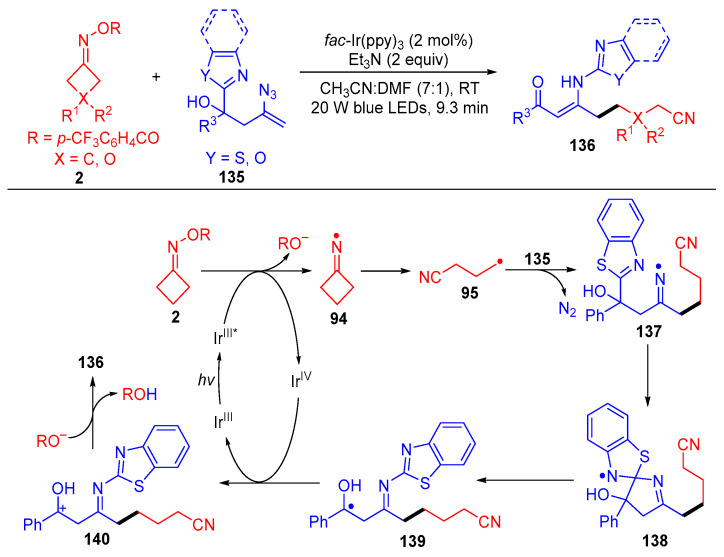
Photoinduced cyanoalkylation of azidyl homoallylic alcohol with cycloketone oxime esters.

Guo, Qiu and co-workers reported a convenient room-temperature synthesis of cyanoalkyl-containing *β*-enamino ketones from heterocyclic-substituted azidyl homoallylic alcohol precursors under visible-light irradiation (Scheme 21) [75]. The remarkable feature is the short reaction time within 9.3 min; yet, the yields of the substrates are generally low, especially for the cyclic ketoxime substrates, which generally have only 20–50% yields. Mechanistically, the addition of the cyanoalkyl radicals **95** to azide olefins **135**, with removal of one molecule of nitrogen, generates the imino radicals **137**, which undergo a 5-*exo* intramolecular radical process to give the key short-lived spiro radical **138**. Subsequently, the spirocyclic radical undergoes C–C bond breaking to obtain the alcohol radical intermediate **139**, which is oxidized by the Ir^IV^ to lose a proton to obtain the final product **136**.

**Scheme 22:**
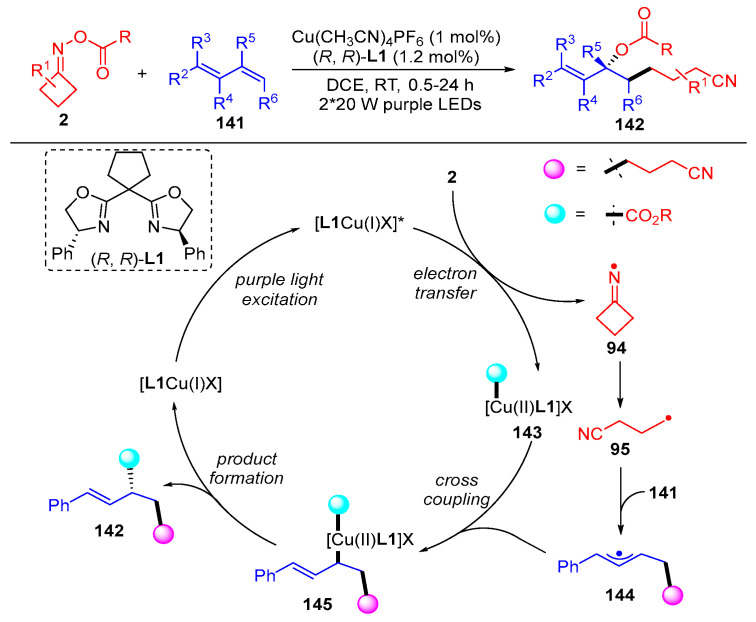
Photoinduced copper-catalyzed asymmetric difunctionalization of 1,3-dienes with cycloketone oxime esters.

The unpaired catalytic synthesis of chiral ligands with transition metals is a hot topic, but also a difficult area in the field of organic synthesis. Chen’s group has developed a purple-light-irradiated enantioselective difunctionalization of 1,3-butadienes using cyclic ketoxime ester as a bifunctional reagent under the action of copper catalysis and ligand (*R*, *R*)-**L1** to generate diversely substituted allylic esters with the ee up to 95% (Scheme 22) [76]. As proposed by the authors, the copper catalyst is combined with the ligand (*R*, *R*)-**L1** to obtain the [**L1**Cu(I)X] species, which is subsequently excited by purple light into the excited state [**L1**Cu(I)X]*. The excited state [**L1**Cu(I)X]* undergoes electron transfer with the cyclic ketoxime ester **2** to generate an imino radical **94**, carboxylate anions and [**L1**Cu(II)X] species. At the same time, the carboxylate anions combine with [**L1**Cu(II)X] to obtain a copper–nucleophile complex **143**. The imino radical generates the cyanoalkyl radical **95** and then attacks the terminal double bond of 1,3-butadiene **141** to obtain the radical intermediate **144**, which is cross-coupled with the above copper–nucleophile complex **143** to provide the intermediate **145**. Finally, the enantioselective generation of sp^3^ C–O bonds provides the expected products **142** and [**L1**Cu(I)X] species, which then participate in the next catalytic cycle.

**Scheme 23:**
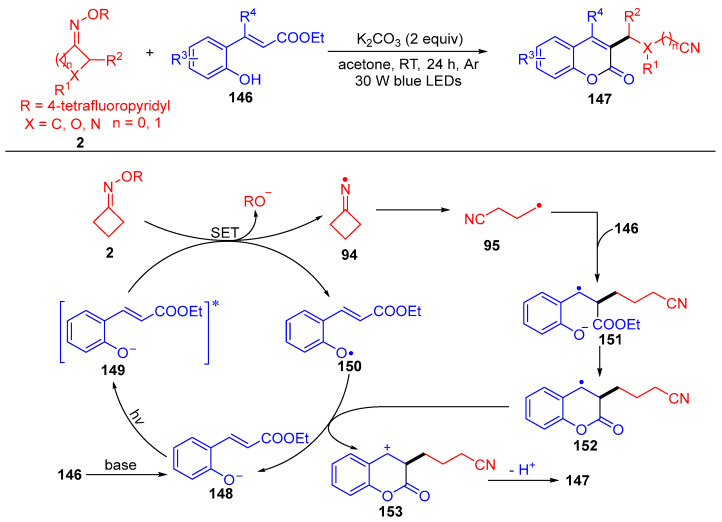
Visible-light-driven cyanoalkylation of 2-hydroxyalkenyl esters with cycloketone oxime esters.

The strategy for visible light-mediated synthesis of the 3-cyanoalkyl coumarin skeleton **147** without an external photocatalyst was developed by Yang and co-workers (Scheme 23) [77]. The *ortho*-hydroxycinnamic esters **146** in the strategy served as both the reactant and photosensitizer. This metal-free cyanoalkylation of cycloketone oxime esters **2** with 2-hydroxyalkenyl esters **146** features a broad substrate scope and good functional group compatibility; especially, the 5-membered oxygen-heterocyclic ketoxime ester could also deliver the target product with a 31% yield.

**Scheme 24:**
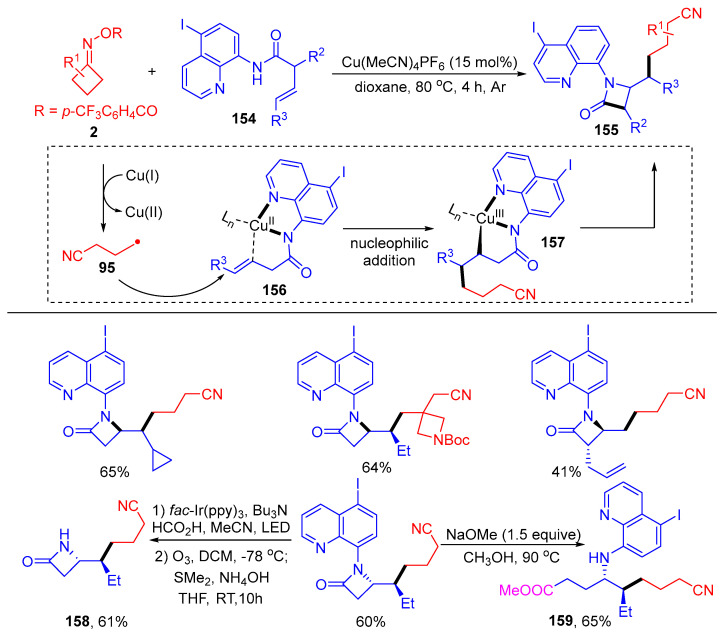
Cu-mediated cyanoalkylation/cyclization of inactivated alkenes with cycloketone oxime esters.

In 2021, He’s group reported a straightforward approach to synthesize *β-*lactam derivatives **155** by using cycloketone oxime esters **2** as the cyanoalkyl radical source via Cu-mediated cyclization of inactivated alkenes containing aminoquinoline **154** (Scheme 24) [78]. In particular, the copper catalyst in the cyclization reaction needs the assistance of the aminoquinoline ligand. Mechanistically, as described in Scheme 24, the Cu(II) catalyst chelated with the substrate **154** to obtain the chelated Cu(II) alkene complex **156**, which will be nucleophilically added by the cyanoalkyl radical **95** to obtain the five-membered Cu(III)-metallacycle **157**. Finally, the *β-*lactam product and Cu(I) are generated through intramolecular reductive elimination. In addition, the authors further transformed the obtained *β-*lactam derivatives **155**, such as by removing the substituent group on the N atom to obtain *β-*lactam **158** and opening the quaternary ring to obtain a long-chain amino ester compound **159**.

#### 3.1.2. Ring Opening and Addition to Alkynes

**Scheme 25:**
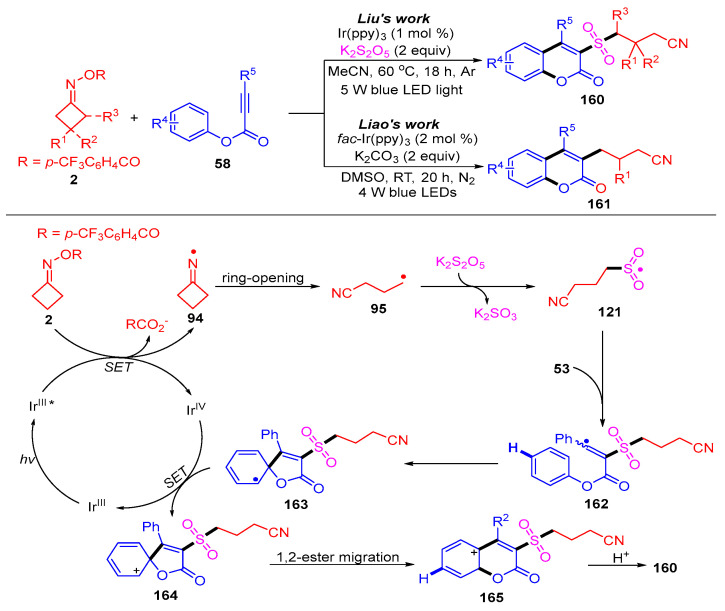
Visible-light-induced cyanoalkylation of alkynoates with cycloketone oxime esters.

The process involving the ring-opening of cyclic ketoxime esters to afford cyanoalkyl radicals and then capturing sulfur dioxide is considered as an effective sulfonylation strategy. Compared with the traditional sulfonylation strategy, it has the advantage of mild and environmentally benign conditions [79,80]. In 2021, Liu’s group demonstrated a cyanoalkyl-radical-triggered cyanoalkylsulfonylation and cyclization transformation of phenyl alkynoates **58** under visible-light conditions to access 3-cyanoalkylsulfonyl-substituted coumarins **160** (Scheme 25) [81]. In this strategy, cyanoalkyl radicals capture sulfur dioxide produced by potassium sulfite to the cyanoalkylsulfonyl radical **125**, and then attack the C–C triple bond of the alkynoates **58** to obtain the intermediate **162**. Generally, there are two different reaction sequences after the spirane structure **163** is obtained by the *ispo*-cyclization of the intermediate **164**. One is that the helix intermediates will be oxidized directly into carbocations before 1,2-ester migration; the other is that the helix intermediates will be oxidized into carbocation after 1,2-ester migration in the form of free radicals. Both approaches are feasible. It is worth mentioning that in the following year, Liao and co-workers developed a SO_2_-free strategy with the same raw material, and the reaction mechanism was believed to be the oxidation after 1,2-ester migration [82].

**Scheme 26:**
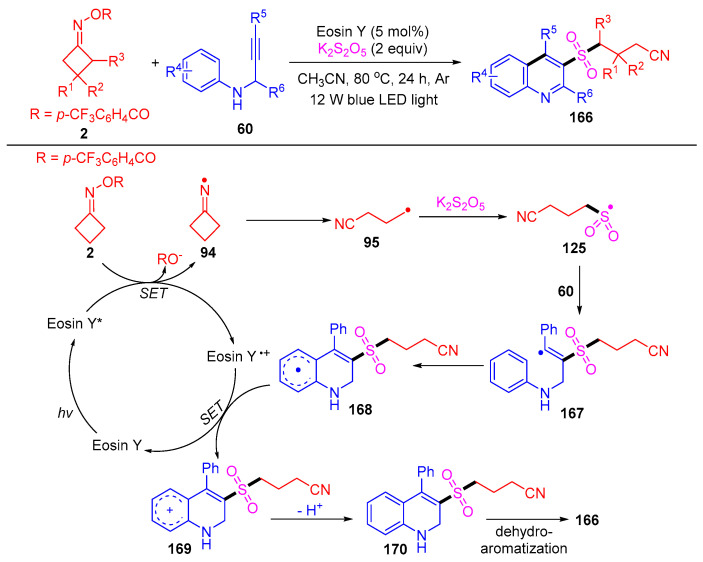
Visible-light-induced cyanoalkylsulfonylation of *N*-propargyl amines with cycloketone oxime esters.

A visible-light-induced, multi-component cascade cyanoalkylsulfonylation of *N*-propargyl aromatic amines **60** with cycloketone oxime esters **2** was discovered by Zhou’s group to synthesize cyanoalkylsulfonylated quinolines **166** (Scheme 26) [83]. This methodology provides a facile and mild route for the synthesis of a large number of cyanoalkylsulfonylated quinoline derivatives, with excellent isolated yields ranging from 53% to 99%.

**Scheme 27:**
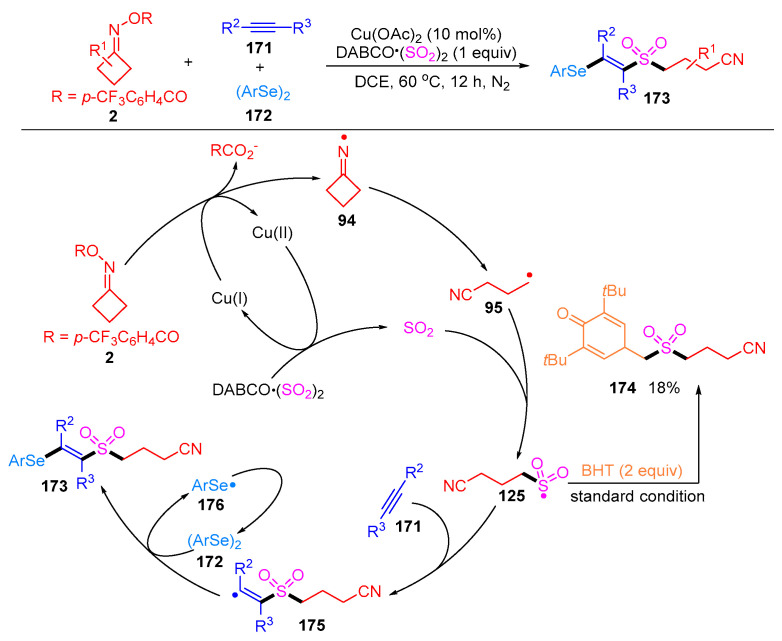
Copper-catalyzed four-component selenosulfonylation of alkynes, cycloketone oxime esters, DABCO and diselenides.

In 2021, Wu et al. established the four-component, free radical, tandem selenosulfonylation of alkynes with cyclobutanone oxime esters **2**, DABSO and diphenyl diselenides **172** for the synthesis of *β*-cyanoalkylsulfonylated vinyl selenides **173** under mild reaction conditions (Scheme 27) [84]. The radical capture experiments demonstrated that the reaction involved a radical course, where cyanoalkylsulfonyl radicals were probably produced. The multicomponent tandem reaction sequentially undergoes the generation of imino radicals **94**, C–C bond breaking to produce cyanoalkyl radicals **95**, sulfur dioxide capture, addition of cyanoalkylsulfonyl radicals **125** to alkynes **171** and coupling of aryl selenyl radicals **176** with alkenyl radicals **175**.

**Scheme 28:**
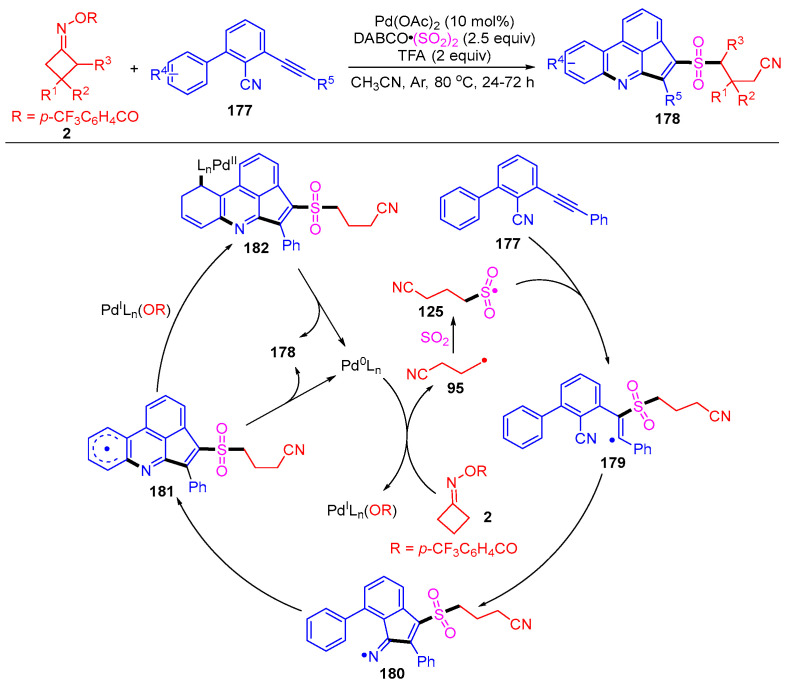
Palladium-catalyzed cyanoalkylsulfonylation of alkynes with cycloketone oxime esters and DABSO.

In the following year, Zhou’s group developed a novel radical tandem cyanoalkylsulfonylation reaction of 3-arylethynyl- [1,10-biphenyl]-2-carbonitriles **177** by using DABSO as the sulfur dioxide source for the synthesis of cyanoalkylsulfonyl-substituted cyclopenta [gh]phenanthridines **178**. (Scheme 28) [85]. Mechanistic studies showed that the alkenyl radical intermediate **179**, obtained by the addition of a cyanoalkylsulfonyl radical **125** to the carbon–carbon triple bond of substrates **177**, undergoes intramolecular cyanogenic cyclization to give the imino radical species **180**, which then adds to the benzene ring to get the conjugated radical **181**. Finally, the radical **181** could undergo two different pathways to provide the final products, i.e., base-promoted homogeneous aromatic substitution and organometallic-based *β*-hydride elimination.

**Scheme 29:**
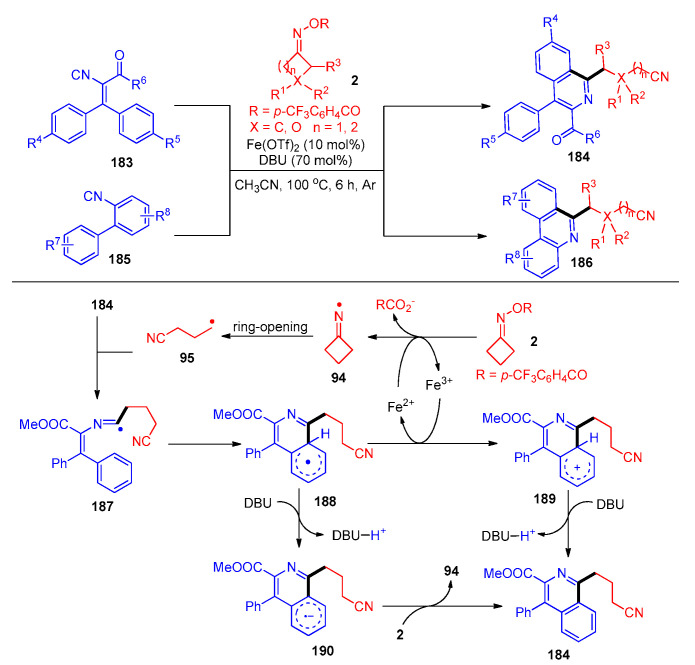
Iron-catalyzed radical cascade cyclization of isocyaniness with cycloketone oxime esters.

Isocyanic compounds are an excellent class of radical acceptors. Some pre-functionalized isocyanic substrates are often used to construct various polycyclic nitrogen-containing heterocyclic compounds. Shao’s group achieved facile construction of a series of 1-cyanoalkyl isoquinolines **183** and 6-cyanoalkyl phenanthridines **185** by cyanoalkylation under iron catalysis using various isocyanines (**184** and **186**) as substrates and cyclic ketoxime esters **2** as radical precursors (Scheme 29) [86]. As shown in the proposed mechanism, the radical intermediate **188** has two pathways to obtain the final product: oxidation by Fe^3+^ species followed by deprotonation by 1,8-Diazabicyclo [5.4.0]undec-7-ene (DBU) or otherwise direct oxidation by oxime ester **2** after deprotonation.

### 3.2. Radical Cross-Coupling

**Scheme 30:**
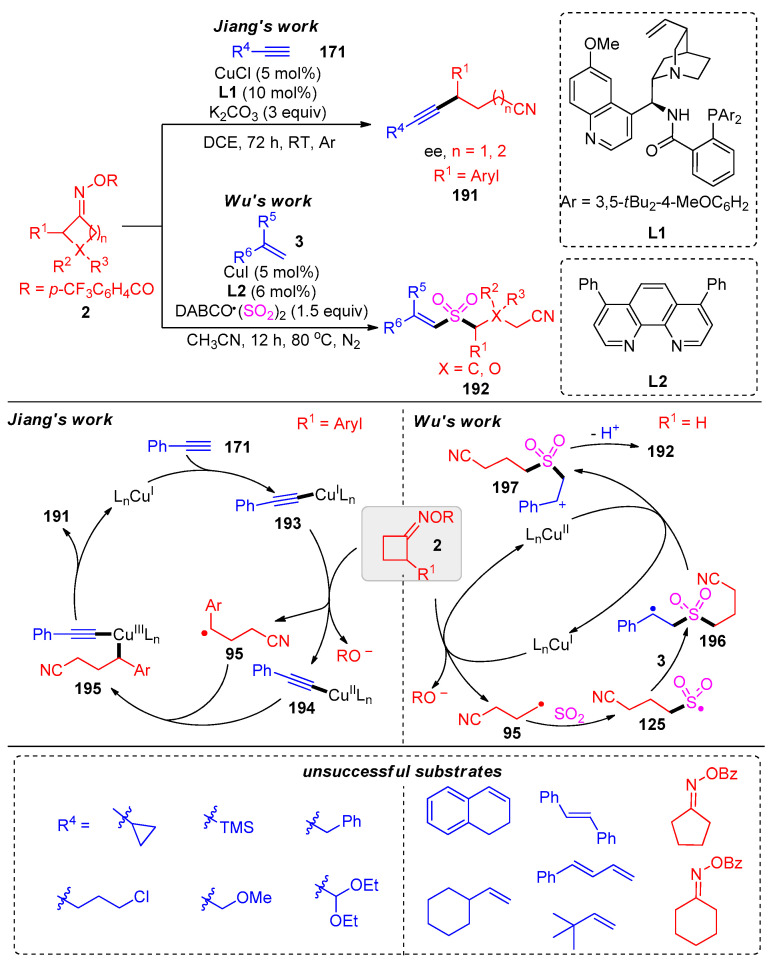
Copper-catalyzed radical cascade of inactivated alkynes or alkenes with cycloketone oxime esters.

In 2021, Jiang and co-workers reported an asymmetric deconstructive alkynylation reaction that is facilitated by copper/chiral cinchona alkaloid-based *N*,*N*,*P*-ligand catalysts **L1** (Scheme 30) [87]. A variety of chiral molecules with cyanoalkyl groups **191** were successfully obtained under mild conditions, including *δ*-alkynyl amides, *ε*-alkenyl carbamate and *ζ*-keto nitrile, with ee up to 99%. The strategy has good functional group tolerance and substrate adaptability, and a range of cyclic oximes, including five- and six-membered reactants, coupled smoothly in moderate to excellent yields. However, this protocol only applies to aryl-substituted alkynes; other alkynes, including ether-substituted and alkyl alkynes, did not react. In addition, Wu’s group also realized a cyanoalkylation strategy with olefins instead of alkynes under copper catalysis [88]. A range of diverse (*E*)-cyanoalkylsulfonyl alkenes **192** were accessed with excellent yields by using copper iodide as the catalyst. According to the available literature and control experiments, the cyanoalkyl radical and cyanoalkylsulfonyl radical were believed to be the key intermediates. Also, the protocol has some limitations in that cyclic olefins, alkyl-substituted olefins, 1,2-disubstituted olefins and 1,3-butadiene olefins failed to give the target products, and five- and six-membered cyclic ketoxime esters did not work in the conversion.

**Scheme 31:**
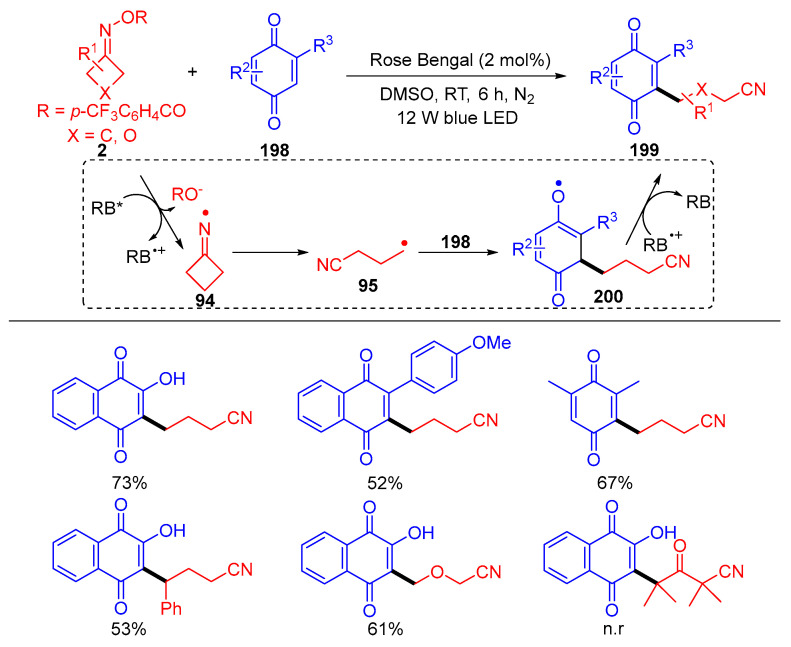
Visible-light-induced cross-coupling of 1,4-quinones with cycloketone oxime esters.

In 2022, a radical tandem functionalization of 1,4-quinones **198** through cyanoalkylation was revealed by Akondi and Mainkar’s group to afford a series of cyanoalkylated quinone derivatives **199** at room temperature in the absence of a transition metal catalyst (Scheme 31) [89]. A range of substituted 1,4-quinones **198** smoothly served as the coupling partners with cycloketone oxime esters **2**, in the presence of 2 mol% Rose Bengal, affording cyanoalkylated quinone derivatives **199** in moderate yields.

**Scheme 32:**
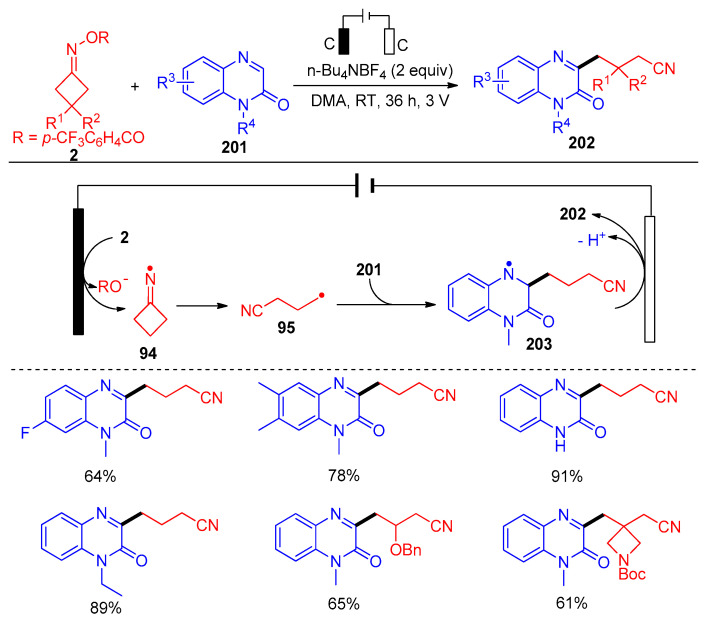
Electrocatalytic C-H cyanoalkylation of quinoxalin-2(1*H*)-ones with cycloketone oxime esters.

The electrochemical technique has long been touted as an environmentally beneficial method due to its inherent ability to achieve redox reactions in a sustainable manner [90,91]. Very recently, Wang’s group developed a pioneering case of electrocatalytic, quinoxalin-2(1*H*)-ones **201**, C-H direct cyanoalkylation that uses cyclic ketoximes **2** as a cyanoalkyl source to successfully construct a series of 3-cyanoalkyl-substituted quinolones **202** (Scheme 32) [92]. Similar to thermal and photochemistry, cyclic ketoxime esters **2** first undergo a single electron transfer at the anode to remove the ester group, and then *β*–C–C bond cleavage occurs to give the cyanoalkyl radical, which then attacks the C–N double bond of the quinoxalin-2(1*H*)-ones to produce the nitrogen radical intermediate **203**. Finally, the intermediate **203** undergoes a single electron oxidation at the cathode, followed by deprotonation, to produce the final cyanoalkylated product.

**Scheme 33:**
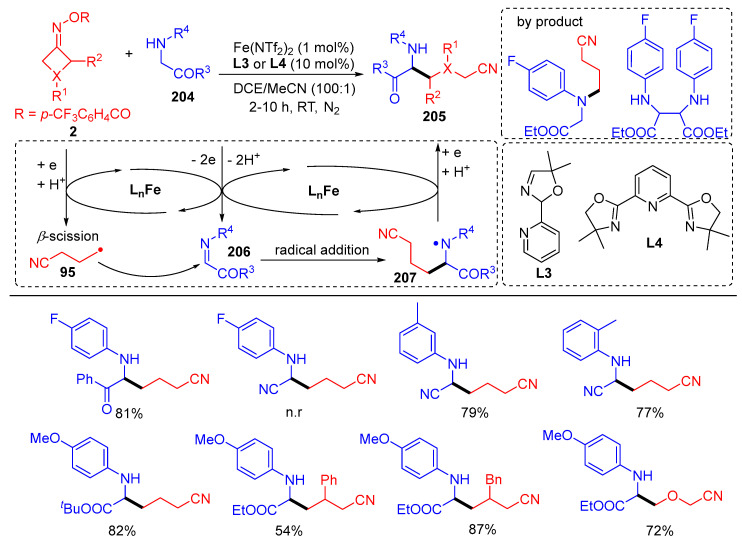
Iron-catalyzed cyanoalkylation of glycine derivatives with cycloketone oxime esters.

An interesting iron-catalyzed selective functionalization process for the direct C_(sp3)_-H cyanoalkylation of glycine derivatives promoted by pyridine-oxazoline ligands was developed by Gong and co-workers (Scheme 33) [93]. A range of substituted glycine derivatives **204** served as cross-coupling partners with cyclobutanone oxime esters **2**, in the presence of 1 mol% Fe(NTf_2_)_2_ and 10 mol% pyridine-oxazoline ligands (**L3** or **L4**), to access cyanoalkylated amino acids and peptides **205** in moderate to high yields. Importantly, this is the first example of C_(sp3)_-H cyanoalkylation with cyclic ketoximes, providing a new pathway for the synthesis of unnatural amino acids. The authors demonstrated experimentally that the interaction between the iron-catalyst and glycinate substrates could greatly improve the catalytic efficiency, and the in situ-formed imine intermediates are a key for high chemo-selectivity.

**Scheme 34:**
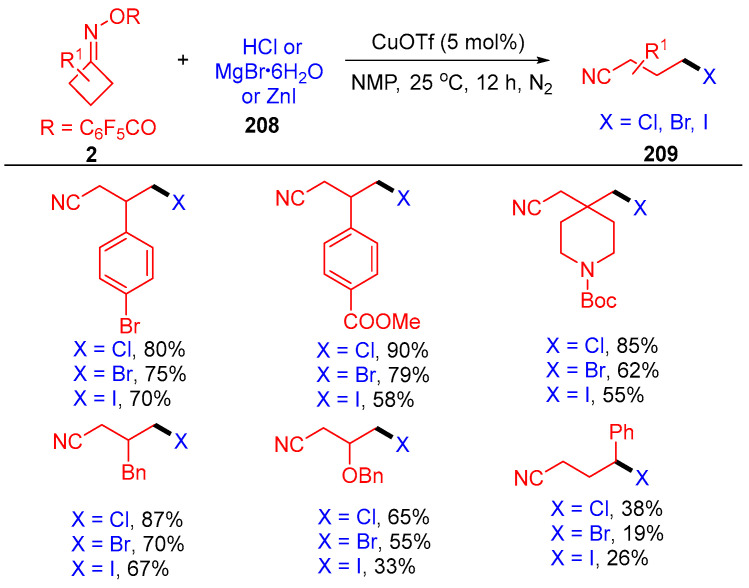
Copper-catalyzed halogenation of cycloketone oxime esters.

Guo and co-workers reported an alkoxy, radical-triggered, ring-opening halogenation of cyclopentyl hydroperoxides under a simple and effective copper catalytic system involving redox-neutral conditions and green halogen sources [94]. In addition, the authors applied this halogenation reaction system to the ring-opening halogenation of cyclic ketoximes **2**, and the structurally diverse halogenated cyanides **209** were constructed in moderate yields (Scheme 34).

**Scheme 35:**
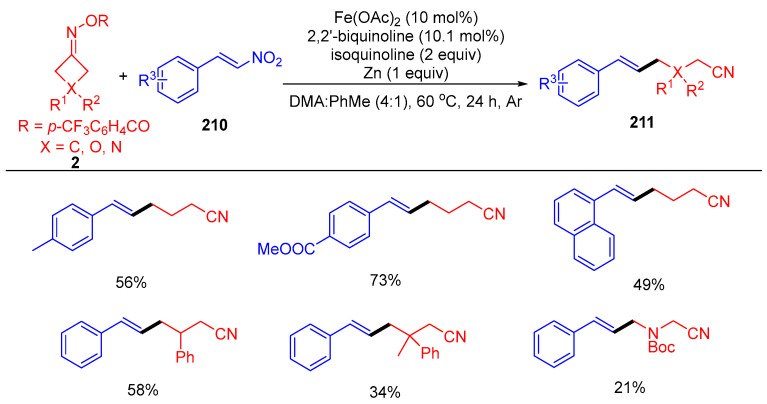
Iron-catalyzed denitrative cyanoalkylation of nitroalkenes with cycloketone oxime esters.

Recently, Qin and co-workers utilized transition metal-catalyzed denitrogenative cyanoalkylation of nitroalkenes by a reductive C–C bond formation process (Scheme 35) [95]. The mild method provides convenient catalytic access to cyanoalkylated styrene derivatives with good functional group tolerance. A variety of cyanoalkylated styrenes were synthesized in moderate to excellent yields.

**Scheme 36:**
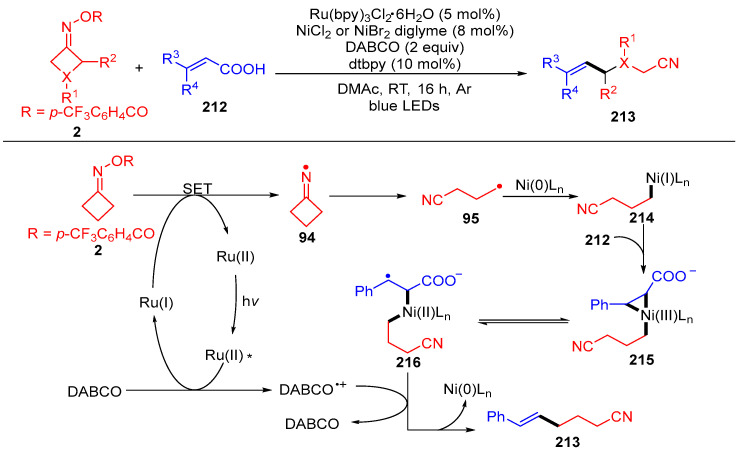
Photo-nickel dual-catalyzed cyanoalkylation of *α, β*-unsaturated carboxylic acids with cycloketone oxime ester.

In 2021, Lu and co-workers reported the decarboxylative cross-coupling of *α,β*-unsaturated carboxylic acids **212** with cycloketone oxime esters **2**, exploiting a photo/nickel dual catalysis (Scheme 36) [96]. Different from the previous decarboxylation mechanism of *α,β*-unsaturated carboxylic acids, this reaction involves the nickelacyclopropane intermediate **215**, which then undergoes decarboxylative elimination to access the final product. This method not only constructs a large number of cyanoalkyl olefins with good functional group tolerance, but also has an important role in the modification of complex molecules.

### 3.3. Radical Coupling

**Scheme 37:**
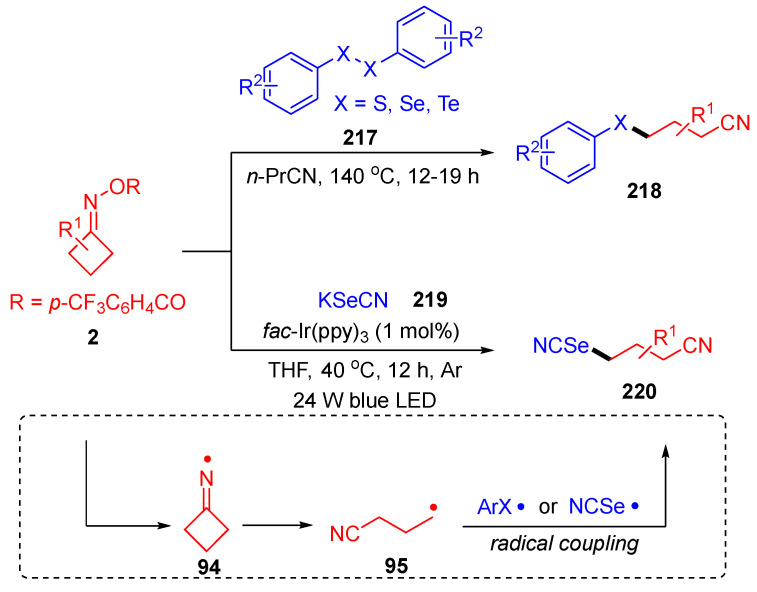
Free radical coupling of cycloketone oxime esters with elements of the group of sulfur.

Elements of the group of sulfur, such as S, Se and Te, are often introduced into the skeletons of various organic compounds as a class of heteroatoms, because organic compounds containing such heteroatoms have a wide distribution and application in pharmaceuticals, drug candidates, agrochemicals, catalysis and functional materials [97]. In 2021, Zhao’s group developed two different chalcogenation reactions of cyclic ketoxime esters **2** (Scheme 37). The ring-opening radical coupling of cyclic ketoxime esters **2** with dichalcogenides **217** occurs under additive-free conditions [98]. The mild photocatalysis enabled its coupling with potassium selenocyanate **219** [99].

## 4. Conclusions

With the increasing requirements for environmental protection, great efforts have been made into advancing green technologies and sustainable chemistry [100,101]. The strategies involving free radical chemistry have been demonstrated as a powerful tool, with features regarding ease of operation, high efficiency, mild conditions and environmental friendliness [21,100,101,102]. Among them, nitrogen-centered radicals are highly versatile reactive intermediates, thus widely exploited for synthetic utility in C–N bond-forming reactions [35]. Readily accessible oxime ester derivatives have gained fame due to their specific N-O structures and their tendency to generate nitrogen-centered radicals with the aid of transition metals or/and photocatalysis.

This paper summarizes related radical reactions of oxime esters through imino radicals, which experience carbon–carbon bond rupture for conversion to carbon-centered radicals (CCRs). Acyl oxime esters and cyclic ketoxime esters are representative substrates of these reactions, which formally include radical addition reactions, cross-couplings, radical couplings, etc. Although the research on oxime esters has been developed rapidly and significant results have been achieved, there are still demands to develop new models of reactivities and expand their applications in related fields. Specifically, the ester group fraction of ketoximes was generally released to form acid by-products, which leads to significant issues of atom economy. Moreover, only a few examples of asymmetric synthesis were disclosed, mostly limited to transition metal catalysis or photocatalysis. In the context of sustainable catalysis, we look forward to further progress in the application of oxime esters with novel reactions and as a multipurpose development in academic and industrial settings.

## Data Availability

Not applicable.
